# Multi-level deep Q-networks for Bitcoin trading strategies

**DOI:** 10.1038/s41598-024-51408-w

**Published:** 2024-01-08

**Authors:** Sattarov Otabek, Jaeyoung Choi

**Affiliations:** School of Computing, Gachon University, Seongnam, 13120 Republic of Korea

**Keywords:** Reward, Social neuroscience

## Abstract

The Bitcoin market has experienced unprecedented growth, attracting financial traders seeking to capitalize on its potential. As the most widely recognized digital currency, Bitcoin holds a crucial position in the global financial landscape, shaping the overall cryptocurrency ecosystem and driving innovation in financial technology. Despite the use of technical analysis and machine learning, devising successful Bitcoin trading strategies remains a challenge. Recently, deep reinforcement learning algorithms have shown promise in tackling complex problems, including profitable trading strategy development. However, existing studies have not adequately addressed the simultaneous consideration of three critical factors: gaining high profits, lowering the level of risk, and maintaining a high number of active trades. In this study, we propose a multi-level deep Q-network (M-DQN) that leverages historical Bitcoin price data and Twitter sentiment analysis. In addition, an innovative preprocessing pipeline is introduced to extract valuable insights from the data, which are then input into the M-DQN model. A novel reward function is further developed to encourage the M-DQN model to focus on these three factors, thereby filling the gap left by previous studies. By integrating the proposed preprocessing technique with the novel reward function and DQN, we aim to optimize trading decisions in the Bitcoin market. In the experiments, this integration led to a noteworthy 29.93% increase in investment value from the initial amount and a Sharpe Ratio in excess of 2.7 in measuring risk-adjusted return. This performance significantly surpasses that of the state-of-the-art studies aiming to develop an efficient Bitcoin trading strategy. Therefore, the proposed method makes a valuable contribution to the field of Bitcoin trading and financial technology.

## Introduction

Trading, which is one of the oldest practices in the economic history of humankind, has undergone significant transformation with the advent of modern technology. Traditional trading, which is often characterized by human discretion and judgment, has been the bedrock of market transactions for centuries. Brokers and traders around the world make decisions based on their understanding of market movements, historical trends, and industry news. However, in the past few decades, fueled by advancements in computing technologies and the rise of data analytics, there has been a paradigm shift from traditional trading methods to algorithmic trading. Algorithmic or algo trading leverages complex mathematical models and algorithms to enable high-speed trading decisions. This method offers a multitude of advantages over traditional trading, such as increased speed and accuracy, reduced costs, and elimination of human emotional bias. In fact, according to a report by GlobeNewswire^1^, the algorithmic trading market size amassed 14.1 billion USD in 2021 and is expected to expand to 41.9 billion USD by 2030, growing at 12.9% per year.

**Figure 1:**
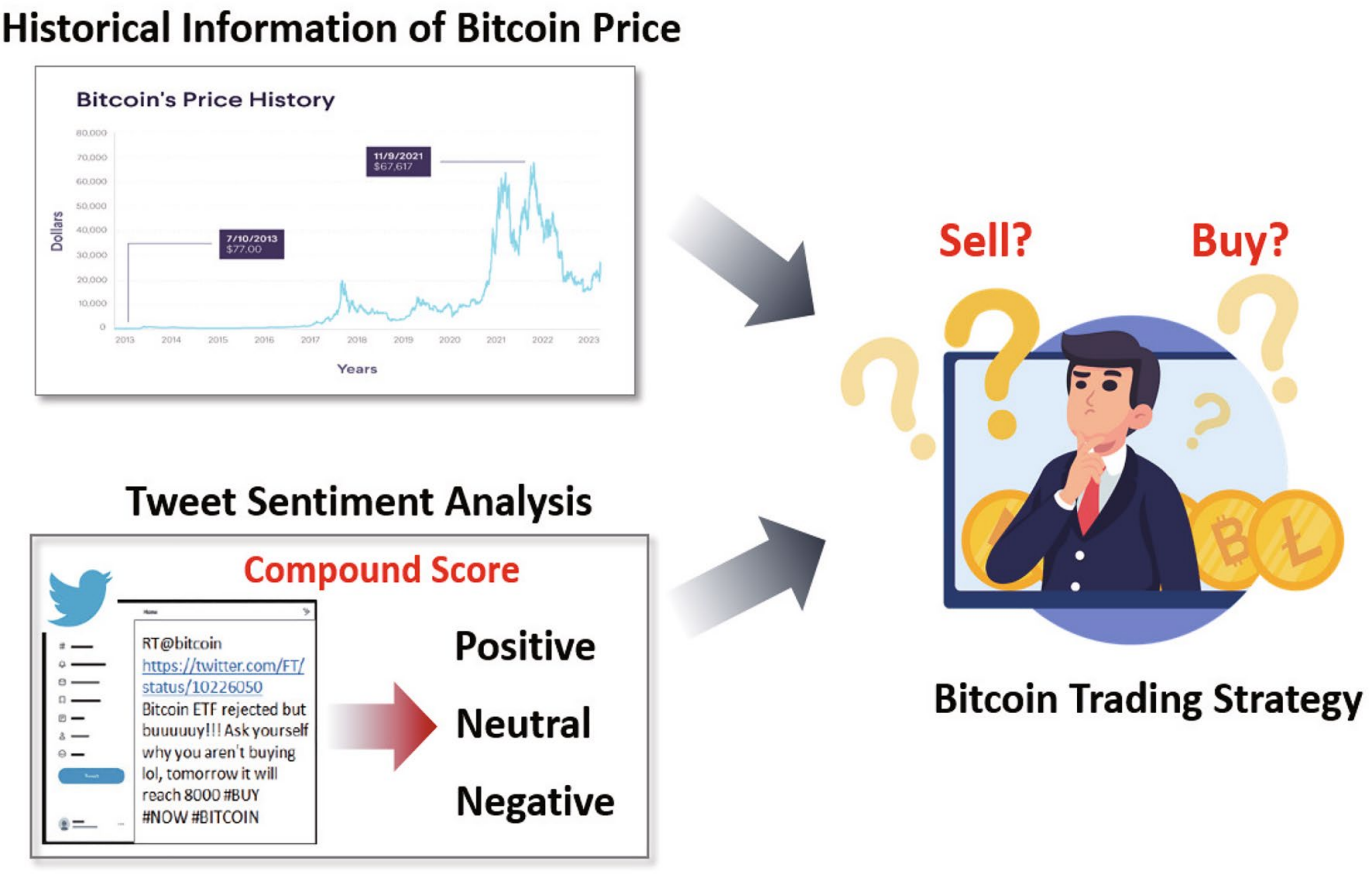
Motivation for Bitcoin trading strategy based on historical information of Bitcoin price and Bitcoin-related tweet sentiment analysis.

This trend toward algorithmic trading has not spared the cryptocurrency market, and the emergence of Bitcoin as a leading digital currency has sparked significant interest among investors and financial traders worldwide^2^. As the pioneer of cryptocurrencies, Bitcoin has opened new venues for trading and investment, thereby revolutionizing the global economic landscape^3^. Bitcoin, with its growing popularity, increasing market capitalization, and decentralized nature, has become a significant player in the world of finance^4^. Its influence extends beyond the realm of cryptocurrencies, affecting traditional financial markets, monetary policies, and regulatory frameworks^5^.

In light of the rapidly evolving cryptocurrency ecosystem, driven by technological advancements as well as increasing public interest, Bitcoin trading has become a critical area of focus for investors seeking to capitalize on its potential for high returns^6^. As the market continues to mature, new opportunities and challenges emerge, requiring innovative methods to navigate the complex and volatile landscape effectively. This necessitates the development of advanced trading strategies that consider multiple factors to achieve optimal performance^7^.

An essential aspect of developing successful trading strategies is to leverage the historical price of Bitcoin^8^. Understanding past trends can help with informed decision-making in real-time trading, aiding in the identification of patterns and market trends that can be exploited^9^. Incorporating historical data into trading algorithms helps establish the context, enabling traders to make informed decisions based on past market behavior^10^. This can lead to more accurate predictions and improved risk management, ultimately contributing to the overall success of trading strategies.

Another crucial component of an effective Bitcoin trading strategy is the integration of people’s opinions on Bitcoin. Social media platforms, such as Twitter, which act as conduits for public opinion, can significantly influence market trends and impact the success of trading strategies^11^. The real-time nature of Twitter enables rapid dissemination of information, leading to potential short-term fluctuations in the market that can be exploited by traders^12^. By incorporating tweet-sentiment analysis into the decision-making process, traders can gain valuable insights into market sentiment, which enables them to anticipate potential price movements and adjust strategies accordingly as shown in Fig. 1.

Active trading, which refers to actual trading with an agent who chooses to buy or sell, is an essential aspect of a successful trading strategy, as it allows traders to take advantage of market fluctuations and price movements^13^. By maintaining a high level of trading activity, investors can capture short-term gains, adapt to changing market conditions, and optimize overall returns^14^. Balancing active trading with profit maximization and risk minimization is a challenging task that has not been adequately addressed in the existing research.

To address these challenges, we adopted the deep Q-network (DQN) technique, which has demonstrated remarkable success in solving complex decision-making problems^15^. DQN offers several advantages over traditional methods, such as an ability to learn and adapt to dynamic environments, handle high-dimensional input spaces, and generalize from past experiences^16^. Moreover, DQN effectively balances exploration and exploitation, which leads to more robust and efficient trading strategies^17^. In particular, the major innovation in this study is that we propose a method to increase learning effectiveness by classifying DQN into several levels. This multi-level structure occurs due to the step of predicting the bitcoin price through Twitter data and the step of the trade through historical price data in order to increase the final trade performance. We call this Multi-level DQN (M-DQN) through the paper. This approach uniquely integrates historical Bitcoin data and Twitter sentiment analysis into our preprocessing pipeline to maximize profits, minimize risks, and encourage active trading in the Bitcoin market. The proposed M-DQN consists of three DQN modules: (1) *Trade-DQN* generates initial trading recommendations by solely relying on Bitcoin historical price data, (2) *Predictive-DQN* is used to obtain future Bitcoin price predictions based on Bitcoin-related tweet sentiment scores and historical price information, and (3) *Main-DQN* explores the synergistic effects of integrating the outputs of the previous two DQN models (trade recommendation and price prediction), thereby examining a combination of these data sources for improved decision-making and trading performance. Furthermore, we also design a novel reward function that encourages the DQN model to focus on these three critical factors, for a more balanced and effective trading strategy.

The M-DQN structure facilitates a more granular approach to learning and decision-making. By compartmentalizing the learning process, each module can specialize and become more efficient in its respective domain. This specialization leads to enhanced performance in each task—be it price prediction, sentiment analysis, or trade recommendation. The decision to integrate these modules into a unified framework is underpinned by the belief that the interplay between different types of data (historical prices and sentiment) can uncover patterns and trading opportunities that might not be apparent when analyzed in isolation. The M-DQN structure is thus not just a sum of its parts but a synergistic ensemble that leverages the strengths of each component to deliver a comprehensive and potent trading strategy.

In summary, we explain the main contributions in some detail, as follows:

(a): First, we propose a preprocessing methodology combining historical Bitcoin price data and Twitter sentiment analysis to extract key market features. Leveraging insights from our previous DQN-based Bitcoin trading model^18^ (Trade-DQN), we analyze over 5 years of Bitcoin’s price history to identify trading signals. We evaluate the influence of public opinion on Bitcoin prices using another DQN-based model (Predictive-DQN), which analyzes sentiments in Bitcoin-related tweets, categorizing them as negative, positive, or neutral, and predicts future price changes in percentage terms.
(b): Second, a novel Main-DQN model is specifically designed for Bitcoin trading. The model incorporates preprocessed data, presenting the opportunity to learn complex patterns and market dynamics. Through extensive training, the Main-DQN model captures intricate relationships between historical prices, sentiment, and market trends. This enables the model to generate an efficient trading strategy capable of identifying and exploiting profitable opportunities in the Bitcoin market.
(c): Third, we propose a novel reward function that considers three important aspects of successful trading: profit maximization, risk minimization, and maintaining active trading. To maximize profit, the reward function encourages the model to identify and exploit profitable trading opportunities. The proposed reward function also penalizes high-risk actions to minimize potential losses, ensuring that the model does not take excessive risks. Further, rewarding a higher frequency of trading actions promotes active trading.
(d): Finally, we provide a comprehensive comparative analysis of our proposed M-DQN method against not only traditional trading strategies but also several recent innovative models. As a result, when compared to traditional strategies that rely solely on raw data, our method demonstrates a remarkable increase in annualized returns by 29.93%, and a 2.74 value in the Sharpe Ratio, a key indicator of risk-adjusted return. Moreover, when we compare these performances with the other contemporary models, the M-DQN outperforms in terms of risk-adjusted value.

The remainder of this paper is organized as follows: “Related work” section provides a review of related works. In “Data preparation” section, the data collection methodology is detailed. “Multi-level DQN” section presents the M-DQN model and the novel reward function. In “Experiment and results” section, the experimental results and performance analysis are explained, highlighting the advantages of the proposed approach over existing methods. In “Discussion” section, the findings of this study, limitations, and potential future research directions are discussed. Finally, “Conclusion” section concludes the paper.

## Related work

This study is related to three strands of literature. First, it contributes to the extensive body of literature on the development of effective trading strategies. Second, it is associated with works that employ Bitcoin historical price data. Third, it is connected to research on the development of trading decisions with a focus on Twitter sentiment data. Table 1 provides general information on related studies, outlining the key algorithms/methods used.

### Exploring Bitcoin trading strategy

The Bitcoin market, with its fast growth, high volatility, and 24/7 availability, has drawn significant individual and institutional investor interest despite the inherent risks of price fluctuations^19,20^. This dynamic, volatile market necessitates advanced trading strategies, leading to the deployment of machine learning as an approach for identifying patterns from historical data and improving trading performance.

Machine-learning algorithms have found wide applicability in financial world problems^21,22^, trading, including portfolio management, risk assessment, and price forecasting^23^. Many studies emphasize predicting futures asset prices, like stocks or Bitcoin^24–27^, underpinning that accurate futures price predictions could guide traders’ decisions, increase profits, and hedge against market risks. Researchers are striving to develop more precise and reliable predictive models that contribute to better trading decisions and financial outcomes. In one of these studies, Attanasio et al.^28^ investigated the application of machine learning methods versus time-series forecasting techniques for predicting subsequent day prices of various cryptocurrencies. In this study, time-series forecasting models yielded a higher average number of trade signals than machine learning-based methods, whereas classification models demonstrated a higher average return per trade than time-series forecasting techniques. In addition, although classification models frequently generated more precise signals, they tended to miss numerous profitable trading opportunities. Slepaczuk et al.^29^ evaluated an algorithmic trading approach using a support vector machine (SVM) model to identify cryptocurrencies with high or low anticipated returns. The SVM strategy ranked fourth in performance, surpassing the S &P B &H strategy, but lagging behind four other benchmark strategies. The authors attributed the SVM model’s modest performance to the large number of required parameters that make it susceptible to overfitting.

A common focus on developing efficient Bitcoin trading strategies by obtaining accurate price predictions are found in existing studies. Kumar et al.^30^ applied various machine learning techniques, including AdaBoost, RandomForest, XGBoost, and Neural Networks, to predict cryptocurrency movements on an intraday scale and to develop a trading strategy. They utilized a diverse range of labels and unique features, such as forecasts from econometric models like GARCH, as well as volume and trade data, to assess their interactions with returns. Their project effectively demonstrated the use of machine learning for trading, framing the problem as a classification task and employing hyperparameter tuning for optimal results. Helder et al.^31^ also investigated the predictability of Bitcoin and the profitability of trading strategies derived from several machine learning algorithms (e.g., linear models, SVM, and random forests). Their positive results further support the robustness of machine learning in terms of the predictability and profitability of Bitcoin, even in challenging market environments.

### Bitcoin historical data in strategy formulation

Historical data consisting of past price movements, trading volumes, and other relevant market information have proven to be valuable resources for traders and investors attempting to develop effective trading strategies that would allow them to make informed decisions in the volatile Bitcoin market^20^.

Numerous studies have shown the potential of using historical data in conjunction with analytical techniques, such as technical analysis and machine learning algorithms. These tools enhance the performance of trading strategies and accuracy of price prediction^32^. For instance, Ciaian et al.^33^ used time-series analysis of daily data from 2009–2014 to investigate the Bitcoin price and its relationship to market fundamentals and investor attractiveness.

In our previous study^18^, we focused on developing an optimal trading strategy using Bitcoin historical price data, employing deep reinforcement learning (DRL) techniques to create a model capable of learning effective strategies through Bitcoin market interactions and adaptive decision-making. Training on historical price data of Bitcoin, Litecoin, and Ethereum, the DRL-based model identified profitable opportunities and managed market risks, demonstrating the potential of DRL in the cryptocurrency market. The research done by Wei et al.^34^ also showed the usage of Bitcoin historical price data can be useful for developing trade planning. Their model capitalized on LSTM’s ability to process historical price data, assisting investors in discerning upcoming market trends. Additionally, they proposed a daily trade strategy model aimed at guiding daily capital management based on market dynamics. This approach is noted for its simplicity, effectiveness, and adaptability to new datasets, marking a significant contribution to quantitative investment strategies and cryptocurrency trading. In order to minimize the risk while maximizing the returns, Hong et al.^35^ also aimed to uncover historical price patterns by developing two models: an ARIMA-based price prediction model (Model 1) and a quantitative trading strategy model using dynamic programming (Model 2). The ARIMA model was used to forecast the trends of gold and bitcoin, providing a foundation for trading decisions. Model 2 utilized the Sharpe ratio as a key parameter to balance investment risk and return. The models were optimized with a particle swarm algorithm, enhancing their efficiency. Notably, the accuracy of the gold and bitcoin price prediction curves was found to be 0.99 and 0.92, respectively, demonstrating the effectiveness of their approach in predicting market trends. Although they aimed for the same goal, our current study however takes into account maintaining the active trading task as well.

Chen et al.^36^ offered insights into the underlying market dynamics by analyzing past price trends. They emphasized the significance of historical Bitcoin price data for forecasting, utilizing machine learning techniques to optimize input data representation and improve prediction accuracy. By comparing various machine learning models, they enhanced the Bitcoin price prediction accuracy, thereby contributing to the development of effective strategies in the cryptocurrency market.

Apart from the role of historical Bitcoin price data, it is essential to explore other factors such as the influence of social media sentiment on trading decisions. In the next subsection, we examine studies that investigate the impact of Twitter sentiment analysis on formulating effective trading strategies, with complementary insight from historical price data to create comprehensive and robust trading strategies.

**Table 1:** Taxonomy of related works.

Categories	Methods
Exploring Bitcoin trading strategy	Q-learning^23^, tree-based classification model^24^, backpropagation neural network^25^, ANN and SVM^26^, RNN and LSTM^27^, forecasting and classification algorithms^28^, SVM^29^, AdaBoost and XGBoost^30^, SVM and random forest^31^, *Deep Q-Network [This paper].*
Bitcoin historical data in strategy formulation	Machine learning and technical analyzing algorithms^32^, time-series analytical mechanisms^33^, deep reinforcement learning^18^, LSTM^34^, ARIMA^35^, random forest, XGBoost, SVM, quadratic discriminant analysis, and LSTM^36^, *Deep Q-network [This paper].*
Twitter sentiment analysis for trading decisions	Recurrent nets and CNN^37^, random forest^38^, linear discriminant analysis^39^, vector autoregression^40^, Q-learning^41^, logistic regression, Naive Bayes, and SVM^42^, Bullish tweet signals^43^, BERT and GRU^44^, FinBERT, CNN, and NLP^45^, *Deep Q-Network [This paper].*

### Twitter sentiment analysis for trading decisions

This subsection explores the role of Twitter sentiment analysis in developing effective trading strategies, highlighting how social media sentiment complements historical price data for a robust strategy. Numerous studies use sentiment data to predict near-future Bitcoin prices, suggesting that the public opinion expressed on social media significantly influences market trends and Bitcoin prices^37–41^. However, few studies have directly incorporated Twitter sentiment data into strategy development, which is a potential growth opportunity for cryptocurrency trading.

For example, Colianni et al.^42^ examined Twitter sentiment analysis of algorithmic cryptocurrency trading strategies. They collected tweets on various cryptocurrencies, including Bitcoin, and preprocessed the data, prior to implementing supervised learning algorithms, such as logistic regression, Naive Bayes, and support vector machines. Sentiment data suggest a trading strategy in which buying or selling decisions are based on tweet sentiments. Their simulation results indicate that the Twitter sentiment-based trading strategy outperforms the naive buy-and-hold strategy in terms of profitability.

Similarly, Gao et al.^43^ studied the impact of financial Twitter sentiment on Bitcoin returns and high-frequency volatility. Their findings showed a significant link between Twitter sentiment and Bitcoin returns, with positive sentiment leading to higher returns and negative sentiment leading to lower returns. Thus, sentiment data can help predict high-frequency volatility in the Bitcoin market. A very similar approach to our current study has been employed by Haritha et al.^44^ by combining historical Bitcoin price data with Twitter sentiment analysis. Their model utilized a Bidirectional Encoder Representations from Transformers (BERT)-based Neural Network for sentiment analysis and a Gated Recurrent Unit (GRU) for price prediction, integrating both user-specific Twitter metrics and historical price trends. This unique amalgamation of sentiment and price data proved effective, with the sentiment analysis achieving a Mean Absolute Percentage Error (MAPE) of 9.45%, and the price prediction showing a notable accuracy with a MAPE of 3.6%. While their study primarily focuses on achieving higher accuracy in predicting Bitcoin prices, our research diverges in its objective. Our study aims not just at predicting price trends but at generating a reliable and actionable trade decision—whether to buy, sell, or hold. Finally, Zou et al.^45^ proposed and back-tested a trading strategy based on several correlated assets, technical indicators, and Twitter content with varying thresholds. They demonstrated that this approach can be used to build a profitable trading strategy with reduced risk compared to a ’hold’ or moving average strategy.

In summary, the studies discussed in this section demonstrate the various methods employed by researchers to develop effective cryptocurrency trading strategies. These studies underline the significance of social media sentiment and Bitcoin historical price data in understanding and predicting market behavior. The studies provide valuable insights and lay the groundwork for further research in this field. Our study builds upon their findings in introducing a unique preprocessing step that combines Bitcoin historical and Twitter sentiment data. This approach potentially creates a more robust trading strategy and contributes to the ongoing efforts to improve the performance of cryptocurrency trading strategies.

## Data preparation

This section presents the data preparation process, which is a crucial step in developing the proposed trading strategy. We consider two types of data: (1) *Bitcoin historical price data*, which indicate the changes in Bitcoin price over a specific period of time and (2) *Twitter sentiment data*, which are Bitcoin-related positive or negative data mentioned on Twitter.

In providing detailed insight into the data preparation stage, the aim is to enhance the transparency and reproducibility of our approach, thereby facilitating the development of accurate trading strategies in the Bitcoin domain.

### Bitcoin historical price data

Bitcoin price dynamics is influenced by a variety of factors ranging from geopolitical events to regulatory changes, making it an intriguing case for an in-depth study. The historical price data of Bitcoin offer a rich tapestry of information that serves multiple purposes and reveals patterns that have emerged over the years, which can be instrumental in predicting futures price movements. This provides insights into market sentiment, shedding light on how markets may react to future events based on past reactions. Additionally, by juxtaposing the data with major global events, certain geopolitical shifts, technological advancements, and regulatory changes that influence the Bitcoin value can be discerned.

**Figure 2:**
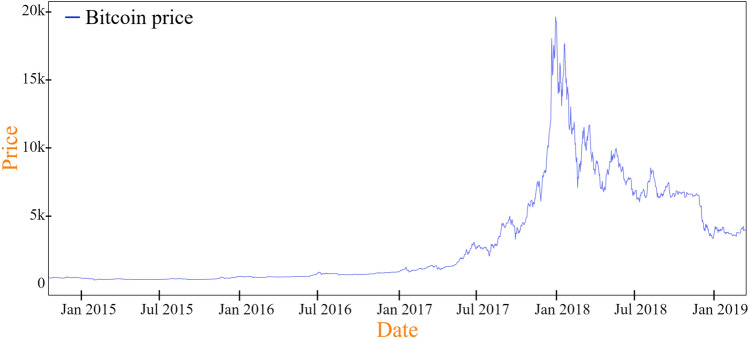
Bitcoin historical price chart graph for the time period of 01.10.2014 and 01.03.2019.

As in our previous work^18^, all historical Bitcoin price data were sourced from the crypto platform^46^ which offers free historical cryptocurrency data, primarily for backtesting strategies and analysis. The historical price data used in this study comprise the closing prices of Bitcoin, spanning the period from October 1, 2014 to March 1, 2019, as shown in Fig. 2. This timeframe was specifically selected because it marks a significant period of substantial price fluctuations for Bitcoin. Such volatility provides a robust testing environment for verifying the effectiveness of the proposed trading strategy. These past fluctuations also serve as a narrative of Bitcoin’s journey. Each spike, dip, and plateau in the price chart indicates a story—a reflection of market sentiment, global events, or technological shifts. Figure 2 traces the trajectory of Bitcoin’s price changes throughout the selected period, offering readers a tangible understanding of its historical evolution.

In essence, the historical price data of Bitcoin go beyond a sequence of numbers, encapsulating the cryptocurrency’s journey and mirroring the market’s evolving sentiments, in responding to a myriad of external influences that have and continue to shape its value. In this study, we aim to decode an intricate sequence of numbers and narratives, offering insights into Bitcoin’s past, present, and potential trajectories.

### Twitter sentiment data

Public sentiment often plays a crucial role in determining asset prices in financial markets, and this effect is pronounced in cryptocurrencies. Social media platforms, particularly Twitter, serve as primary conduits for public sentiments, making them invaluable sources of data for sentiment analyses^47–49^.

This study builds on the methodology presented in previous research, where Twitter sentiment data were employed to predict Bitcoin’s near-term price fluctuations^41^. The primary focus of this endeavor is to analyze the sentiments in tweets pertaining to Bitcoin, for a better understanding of community’s perceptions and potential market movements.

Data collection involved aggregating over seven million tweets related to Bitcoin from April 1, 2014, to November 14, 2018. The extraction utilized Twitter’s streaming API, targeting keywords such as #Bitcoin, #bitcoin, #BTC, and #btc. The detailed statistics for this dataset can be found in Table 2. Given the inherent noise in raw tweets, preprocessing is of paramount importance. The data were subjected to rigorous cleaning, which involved the removal of URLs, unnecessary hashtags, extraneous symbols, and other irrelevant content. On the cleaned dataset, sentiment analysis was conducted using the VADER Python library^50^. The output sentiment scores were further categorized such that scores between − 1 and 0 indicated negative sentiment; a score of 0 was considered neutral; and scores between 0 and 1 indicated positive sentiment. In this study, to indicate the state of DQN, the continuous values were rounded to the second decimal place and used as discrete values.

**Table 2:** Statistical information on dataset.

Definition	Value
Starting time of tweet gathering	01.04.2014
Ending time of tweet gathering	14.11.2018
Number of total tweets	7,142,716
Tweet with keywords used once	4,294,621
Tweet with keywords used twice	1,927,842
Tweet with keywords used more than three times	1,057,181

The aim of integrating Bitcoin historical price data with Twitter sentiment data is to produce a more refined trading strategy. Combining objective historical price data with subjective sentiment data offers a comprehensive approach that can enhance the predictive accuracy of the trading model. In leveraging both datasets, the goal is to develop a trading strategy that accounts for past price movements while also adapting to current public sentiment.

## Multi-level DQN

This section presents the learning algorithm employed to develop an effective trading strategy by leveraging the processed datasets. A more comprehensive understanding of the proposed model is provided in the three subsections. The proposed M-DQN consists of three independent DQN-based models: Trade-DQN, Predictive-DQN, and Main-DQN. The subsections respectively cover the theoretical background of RL and DQN, a summary of the results from the Trade-DQN and Predictive-DQN models, and the design methodology of the proposed Main-DQN model.

### Background—RL and DQN

As mentioned above, because the proposed M-DQN is based on the RL and DQN structures, the basic concepts are introduced prior to describing the method in detail.

#### Reinforcement learning

Reinforcement learning is a machine-learning paradigm in which an agent learns to make decisions by interacting with its environment^51^. The goal of the agent is to maximize its cumulative reward by discovering an optimal policy that maps states to actions. The agent performs actions based on its current state, and the environment responds by providing feedback in the form of rewards or penalties. This process is iterative and continues until the agent has acquired a sufficient understanding of the environment to ensure that the given task is well performed.

The foundation of RL lies in the MDP framework, which comprises a tuple ($S, A, P, R, \gamma$), where S denotes the set of states; *A* represents the set of actions; *P* is the state-transition probability function; *R* is the reward function; and $\gamma$ is the discount factor with $0\le \gamma \le 1$. This indicates the agent’s preference for present over future rewards. When $\gamma$ is closer to zero, the agent prioritizes immediate rewards, whereas a $\gamma$ value of approximately one suggests that the agent values future rewards almost as much as immediate rewards.

The agent’s objective is to learn an optimal policy $\pi$ that maximizes the expected cumulative reward, which is known as the value function, for each state. The value function *V*(*s*) is defined as the expected cumulative reward starting from state *s* and following policy $\pi$. Similarly, the action-value function *Q*(*s*, *a*) represents the expected cumulative reward starting from state *s*, taking action *a*, and following policy $\pi$.

A popular method for solving RL problems is Q-learning, which is a model-free, value-based method that directly estimates the optimal action-value function^52^. Q-learning is an off-policy algorithm that learns the optimal policy regardless of the agent’s current policy. In Q-learning, the agent updates its action-value function using the Bellman equation, which expresses the optimal value of a state-action pair as the immediate reward plus the discounted future value of the next state-action pair. The agent iteratively updates the Q values using this equation until the optimal Q values converge.

#### Deep Q-network

Building upon the foundations of Q-learning, DQN is an extension that combines reinforcement learning with deep learning techniques^15^. It uses a deep neural network as an approximator to estimate the action-value function *Q*(*s*, *a*). DQN addresses the main challenges of traditional Q-learning, such as learning stability. Moreover, by employing deep learning, DQN can handle high-dimensional state spaces, such as those encountered in image-based tasks or large-scale problems^53^.

To ensure stable learning, DQN incorporates two essential techniques: experience replay and target networks. Experience replay is a mechanism that stores an agent’s experiences (i.e., state transitions and rewards) in a replay buffer^54^. During training, the agent samples random minibatches of experiences from the buffer to update the Q values. This process helps break the correlation between consecutive experiences, thereby reducing the variance of updates and leading to more stable learning.

Complementing experience replay, target networks address the issue of moving targets in the Q-value update equation. In DQN, a separate neural network called the target network is used to compute the target Q-values for the Bellman update. The target network has the same architecture as the main Q-network. However, its parameters are updated less frequently, by periodically copying weights from the main network. This technique mitigates the issue of nonstationary targets and improves learning stability.

In summary, RL and DQN provide a robust and scalable framework for learning optimal policies in complex environments with large state spaces. By leveraging deep learning techniques, DQN effectively tackles the challenges of scalability and stability in traditional Q-learning. In the context of this study, the DQN framework was applied to develop an enhanced trading strategy that incorporates both Bitcoin historical price data and Twitter sentiment data.

### Preprocessing DQN

As described previously, the proposed M-DQN consists of two parts: (1) *Preprocessing DQN* and (2) *Main Trading DQN*. Preprocessing DQN is a DQN that preprocesses the input data of Main DQN using the original data. For this purpose, two different types of DQN: trade-DQN with Bitcoin price data and predictive-DQN with Bitcoin price and tweet sentiment data, were constructed to deal with different datasets. A detailed explanation of the preprocessing DQN is provided below.

#### Trade-DQN with Bitcoin price data

**Figure 3:**
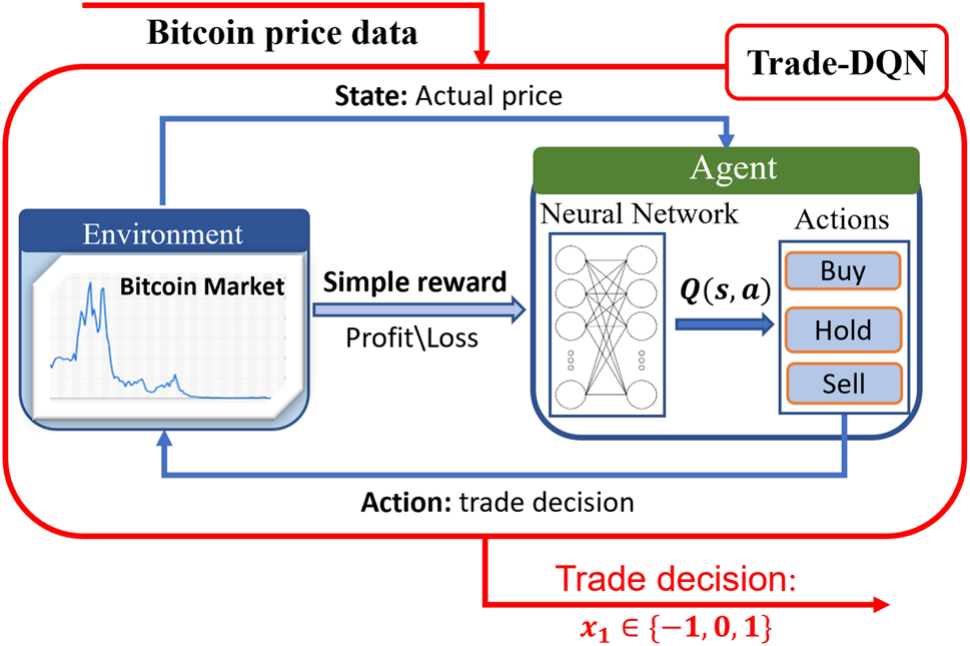
Trade-DQN model structure^18^.

In the Trade-DQN model, the agent attempts to maximize short-term profits in the Bitcoin market, learning from features related to market conditions, relationships between historical Bitcoin prices, and agent’s current financial position. Over time, the agent learns how to make optimal investment decisions—buy, sell, or hold Bitcoin.

The agent interacts with its environment, which is defined as an hourly Bitcoin market. That is, the agent observes the environment and receives hourly Bitcoin price data as a state, chooses an action based on the policies learned during training, and obtains a reward for the actions taken. In this study, the state, action, and reward are denoted by $s_t$, $a_t$, and $r_t$, respectively for all DQN models at time *t*.

In the Trade-DQN step, the state is defined as $s_t:=AP_t$, where $AP_t$ is the actual Bitcoin price at time *t*. The Bitcoin price is considered up to the second decimal place and used as a discrete value. The action of the agent is $a_t \in \{buy, hold, sell\}$ *i.e.,*, the agent can perform three types of actions: buy, hold, and sell Bitcoins, as shown in Fig. 3. Reward $r_t$ is designed to encourage the agent to make profitable trades and discourage unprofitable or indecisive actions. If the agent chooses to “hold,” it gets zero feedback from the environment ($r_t=0$). However, if the “hold” action is repeated consistently several times (more precisely twenty times), the agent is punished with a negative reward ($r_t=-1$ if the number of consecutive “hold” actions $m \ge 20$). After each “sell” action, an agent gets a reward from the environment, negative or positive. The reward value depends on the profitability of the selling action. This is calculated by subtracting the selling price, denoted by $P_{sell}$ from the last purchasing price, denoted by $P_{buy}$, whereby the reward is $r_t=P_{sell}-P_{buy}$. If the agent continuously chooses the “buy” action and the number becomes higher than the limit (in this case 20), the agent receives a negative reward ($r_t=-1$). This is to prevent the market from making many sequential purchases and improve the agent’s performance.

The DQN model consists of four multilayer models designed to suggest one of three possible actions: buy, sell, or hold a position. The first layer has 64 hidden units; the second layer has 32; the third layer has eight neurons; and the last layer contains three units, corresponding to the number of possible actions. The activation function uses a rectified linear unit (ReLU) in the first three hidden layers and a linear function in the last layer. The mean square error (MSE) is used as the error function. The final results of all the four models are used to assess the confidence indicators for each of the three available outcomes.

#### Predictive-DQN with Bitcoin price and tweet sentiment data

**Figure 4:**
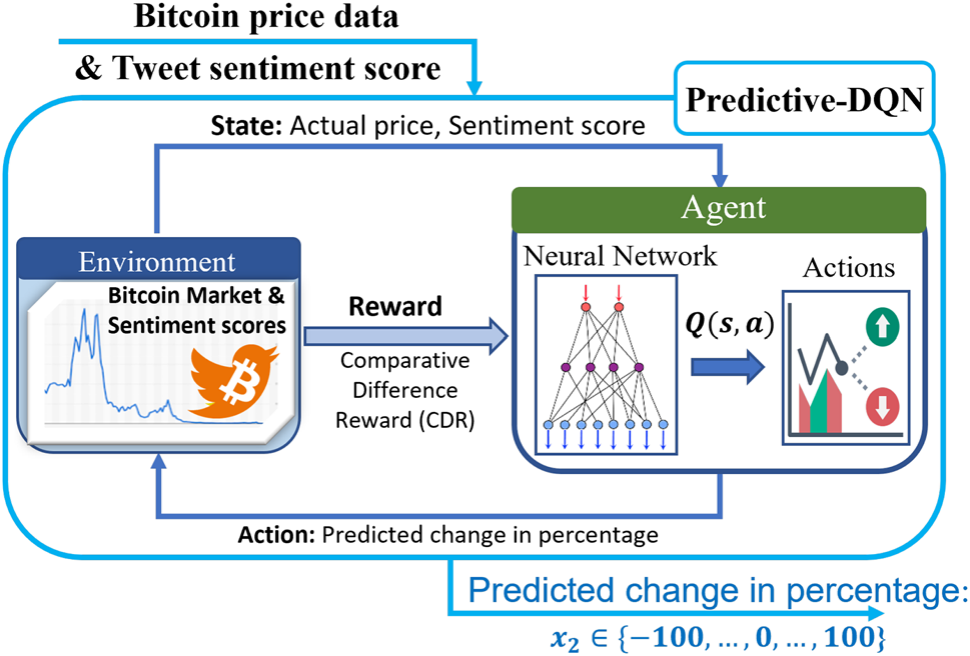
Predictive-DQN model structure.

In Predictive-DQN, Bitcoin-related tweets are utilized to extract sentiments, thereby separating them into positive (compound score between 0 and 1), neutral (score of 0), and negative (score between 0 and − 1) categories as described above. In leveraging these sentiment scores and employing the DQN algorithm, the objective is to construct a model capable of predicting future Bitcoin prices. Hence, in this model, the state $s_t$ is defined by the pair $s_t:=[AP_t, TS_t]$, where $TS_t$ is the Twitter sentiment score at time *t*. Because up to the second decimal place is considered for $AP_t$ and $TS_t$, the state space is discrete. Based on this state, the action of the agent is defined as a number between $a_t \in \{-100, -99, \ldots ,0, \ldots ,99,100\}$, representing the future prediction of the price as the change from its current value in terms of percentage (Fig. 4). The comparative difference reward (CDR) function designed in our previous work^41^ was adapted as the reward function. This is a unique reward function designed to provide more nuanced feedback to the model based on the accuracy of its predictions. The CDR function considers the rate of change in the actual Bitcoin price and introduces the concept of a zero-reward value, which was defined in^41^, as follows:

##### Definition 1

^41^ Let $\alpha = (AP_{t} - AP_{t-1})/AP_{t-1}$ where $AP_t$ is the actual price of Bitcoin at time *t*, and $AP_{t-1}>0$ *i.e.,*the rate of change in the actual price. Let $PP_t$ be the predicted price at time *t* and let $l=AP_t - PP_{t-1}(1+\alpha )>0$. This point is referred to as *zero-value reward* ($ZR_t$) at time *t* where the difference from $AP_t$ is *l*.

**Figure 5:**
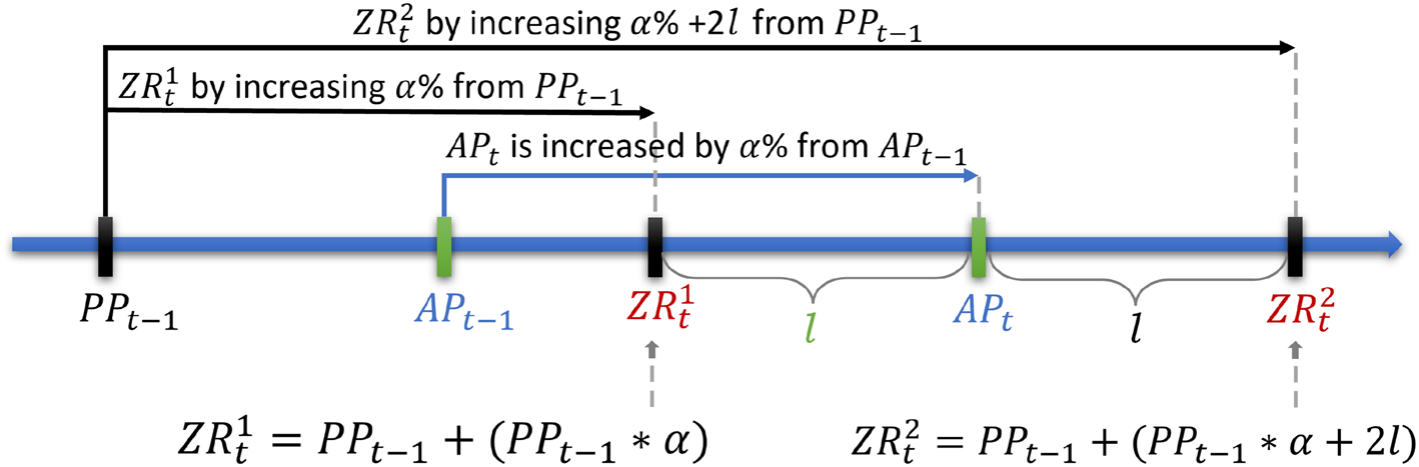
Computation of zero-value reward^41^.

The reward value is then computed based on whether the predicted price ($PP_t$) is higher or lower than the actual price ($AP_t$). Therefore, two ZRs exist, as shown in Fig. 5. The former case is denoted by $ZR_t ^1$ and the latter case by $ZR_t ^2$. If $PP_t$ is smaller than $AP_t$, the agent receives a negative reward ($PP_t < ZR_t ^1$) or a positive reward if $PP_t$ is between $ZR_t ^1$ and $AP_t$. Mathematically, the reward value is calculated as follows:

1 $$\begin{aligned} r_t =\frac{PP_t-ZR_{t}^{1}}{AP_t-ZR_{t}^{1}} *100\% \end{aligned}$$

If $PP_t$ is higher than $AP_t$, the reward is positive if $PP_t$ is between $AP_t$ and $ZR_t ^2$, and the reward is negative if $PP_t$ is higher than $ZR_t ^2$. In this case, the equation for calculating the reward value is:

2 $$\begin{aligned} {r_t} =\frac{PP_t-ZR_{t}^{2}}{AP_t-ZR_{t}^{2}}*100\% \end{aligned}$$

In both cases, the reward increases when the predicted price approaches the actual price. The CDR function provides a more detailed feedback to the model, allowing it to better adjust its predictions over time.

The Predictive-DRL model comprises five multilayer models designed to output any number between − 100 and 100 with up to two decimal-point precision. As previously mentioned, this number represents the percentage change from the actual price. The first layer serves as the input layer and has two neurons that reflect the two features of the Bitcoin price: the actual price and sentiment scores. The three subsequent layers, referred to as the dense layers, contain 64 hidden units each. The final output layer comprises 20,001 units. These units correspond to the number of possible actions, accounting for all possible numbers between − 100 and 100 with up to two-decimal point precision. The ReLU function serves as an activation function for the first three hidden layers, whereas the output layer uses a linear function. MSE was adopted as the error metric.

To summarize, the results from the Predictive-DQN model were positive, achieving 86.13% accuracy and drawing attention to the effects of Bitcoin-related tweets on Bitcoin futures price changes. Therefore, in this study, to develop an efficient Bitcoin trading strategy, a unique dataset was proposed to include market decisions and market prediction information for Bitcoin.

### Main trade recommendation DQN with integrated data

**Figure 6:**
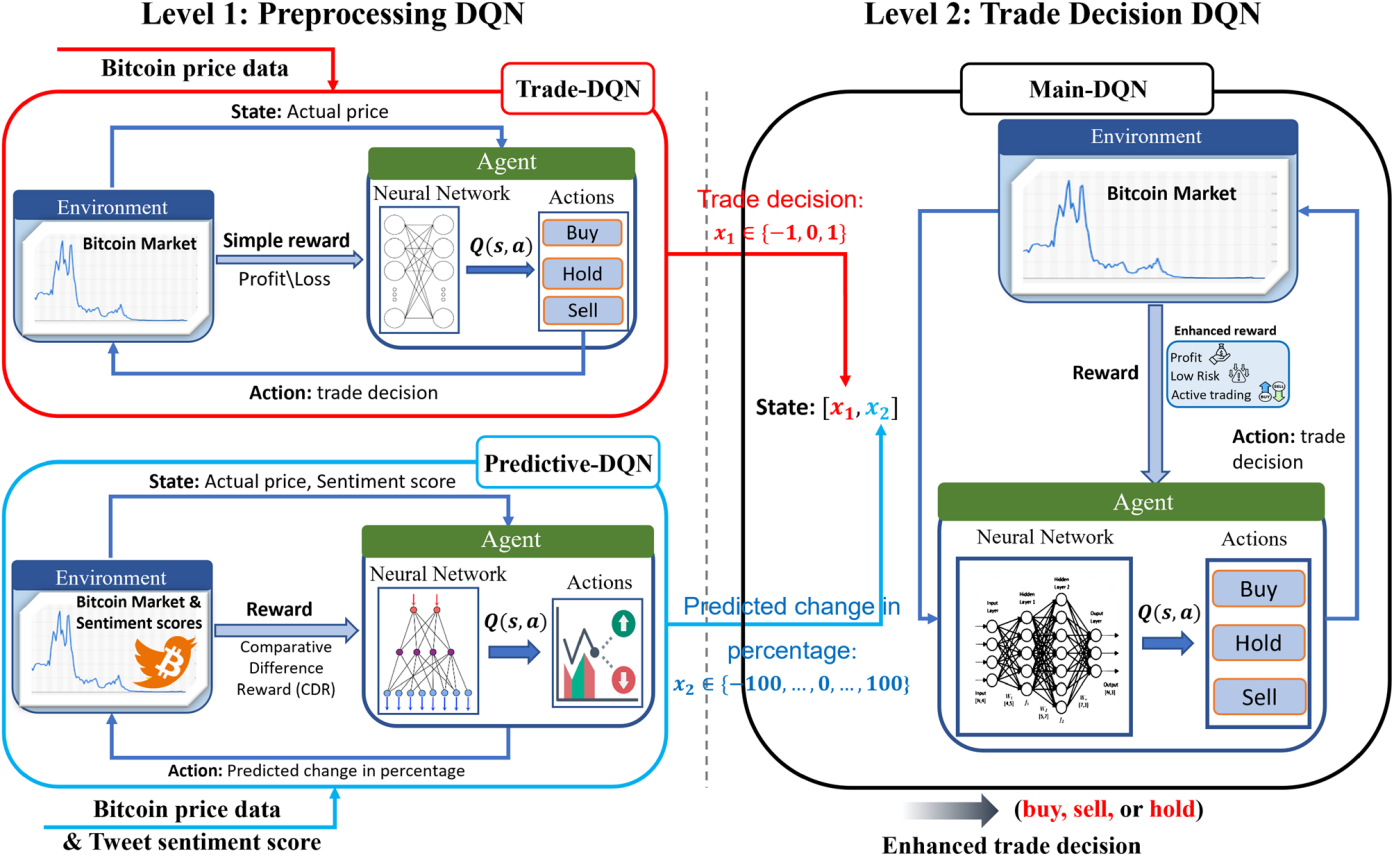
Proposed M-DQN model. In Preprocessing DQN: Trade-DQN receives Bitcoin price data as input, and generates initial trade decisions as output $x_1$; Predictive-DQN receives Bitcoin price data along with Tweet sentiment score as input and generates predicted change in percentage as output $x_2$. In Trade Decision DQN: Main-DQN utilizes the two preprocessed outputs [$x_1,x_2$] as input, thereby generating the final trade decision as output.

In the main DQN, for the final trade, the output data of the aforementioned two Preprocessing DQNs are used for learning. Therefore, the performance of the proposed Main-DQN model is based on the output results from Trade-DQN, which provide trade recommendations, and Predictive-DQN, which offer futures price predictions. Before providing a detailed explanation of the Main-DQN model, the process of leveraging these outputs is described.

#### Data integration

First, two different types of output data are merged into one. Combining these datasets allows us to develop a more comprehensive trading strategy that leverages the strengths of both data sources.

Large-scale timespans were considered for both datasets. As there was a slight difference in the time periods covered by each, to maintain data integrity and consistency, we identified overlapping periods between the two datasets. Furthermore, to satisfy the research objective of uncovering correlations between variables within these datasets, it is crucial that the data originate from a consistent timeframe. This alignment guarantees that genuine relationships are not distorted by variations over time. The overlapping time periods in the datasets were identified as spanning from October 1, 2014 to November 14, 2018, comprising 1505 days. This overlapping period enabled us to effectively combine the datasets and ensure the integrity of the analysis.

Next, the two datasets were merged into a single dataset by aligning trading recommendations and futures price predictions based on their respective timestamps. For each hour within the overlapping period, the corresponding trading recommendations and futures price predictions were placed in the same row. This approach facilitates the seamless integration of data, allowing a more effective examination of the relationship between trading recommendations and futures price predictions.

Given that the objective was to develop an hourly trading strategy, the total number of hours within the experimental period were calculated by multiplying the number of days (1505) by 24 h. This resulted in 36,120 h of data, which formed the basis of our dataset. Therefore, the final dataset comprised 36,120 rows, with each row representing an hour within the experimental period. Each row contained trading recommendations and futures price predictions for a specific hour. This integrated dataset enabled us to explore the synergistic potential of combining Bitcoin historical price data with Twitter sentiment analysis, ultimately aiming to enhance our trading strategy and improve its performance in the volatile Bitcoin market.

#### Modeling the main DQN

After obtaining the integrated dataset, the DQN-based Bitcoin trading model (Main-DQN) was built. Similar to the preprocessing models, the proposed DQN model, which acts as an agent, interacts with the environment represented by the Bitcoin market. The MDP elements of state, action, and reward are defined as follows:

- *State Space* $\mathcal{S}$ A state represents the current situation in the market, which is crucial for making informed decisions. In our study, each row of the prepared dataset describes hourly data of historical price and Twitter sentiment and is considered a state $s_t:=[x_1, x_2] \in \mathcal{S}$ at time *t*. Specifically, each state is a two-dimensional array, where the first element $x_1$ can be either − 1, 0, or 1, indicating a sell, hold, or buy recommendation, respectively, and the second element $x_2$ is a number between − 100 and 100, representing the predicted futures price change percentage based on Twitter sentiment, as shown in Fig. 6.
- *Action Space* $\mathcal{A}$ An action represents the decision made by the agent at a particular state. In our trading task, the action of the agent $a_t \in \mathcal{A}$ at time *t* is defined as the final decision on trading, which can represent one of three options: buy a Bitcoin from the market, hold, or sell. The agent learns how to choose the most suitable action based on the state information and experience, aiming to maximize the expected cumulative reward.
- *Reward Function* *r* For the trading task, the three important aspects considered for effective trading are: gaining high profits, keeping risk at a low level, and maintaining active trading, based on which the proposed reward function evaluates the agent’s decision and guides the learning process. As mentioned earlier, achieving high returns is one of the components of an effective trading strategy. In this study, the agent’s performance is assessed in terms of gains through profit and loss (PnL) calculations after each decision. It is important to note that transaction fees, which are charges incurred by traders when conducting buy or sell actions in the market, play a role in determining the PnL. These fees vary depending on the trading platform; however, they typically range between 0.1 and 1.5% of the trade value^55^. For the purpose of this study, a constant 1.5% transaction fee rate was assumed. Despite the transaction fee, a PnL can only be generated when a trade has both purchasing and selling prices. Therefore, when the agent decides to buy or hold, it receives zero reward, and as soon as it decides to sell, the selling price is subtracted from the sum of the buying price of Bitcoin and all transaction fees. If the resulting value is positive, the agent receives an equivalent positive reward. If the value is negative, the agent receives a penalty equal to the negative value. Thus, the reward function considers both the profit potential and transaction costs involved in trading. To define the reward function mathematically, the following notation is introduced: $P_k^{buy}$ and $P_k^{sell}$ represent the buying and selling price values of the Bitcoin for a given order *k* (i.e., the 1st Bitcoin, 2nd Bitcoin, and so forth), whereas $c_k^{buy}$ and $c_k^{sell}$ refer to the transaction fees incurred during the purchase and sale of the *k*th Bitcoin, respectively. Under these terms, the PnL for each Bitcoin transaction can be computed as:
  3 $$\begin{aligned} {PnL_k} =P_k^{sell} - P_k^{buy} - c_k^{buy} - c_k^{sell}. \end{aligned}$$
  This formula specifies a comprehensive method for quantifying the net profit (or loss) obtained from the *k*th Bitcoin transaction, after considering the transaction fees. This way, the reward value at time-step *t* is equal to the value of $PnL_k$ ($r_t=PnL_k$). In describing the reward function, the second critical factor for an effective trading strategy is to maintain a low risk level. In this work, risk level is described as the percentage of the investment that the model is allowed to risk. After each decision made by the agent, the amount of investment is calculated, and if it is below a certain threshold, the agent receives a penalty. By contrast, if the investment is above the threshold, the agent receives zero reward. More precisely, $I_{current}$ is defined to represent the current value of the investment, which is determined at each time-step *t* after purchasing Bitcoin. This value is computed by deducting all relevant expenses ($P_k^{buy}$ and $c_k^{buy}$) from the initial investment amount, $I_{Initial}$. The threshold, denoted by $\alpha$, represents the maximum permissible part of the investment that the agent is allowed to risk. The final factor for an efficient trading strategy is to maintain active trading, which refers to buying or selling Bitcoins in the market rather than simply holding them. Encouraging the agent to engage in active trading is essential for capitalizing on market opportunities and adapting to changing market conditions. To promote active trading, a threshold is established and monitored to determine the number of active trades at the time of reaching or exceeding this threshold. Let *m* be the sum of all the number of purchases and sells and $\omega$ be the threshold for active trades. In this scenario, the agent receives a negative reward only when the number of active trades (*m*) exceeds the threshold, $r_t = -1,$ if $m > \omega$. Then, the reward $r_t$ can be computed depending on the action $a_t$ by:
  4 $$\begin{aligned} {r_t=} \left\{ \begin{array}{l} -1, \quad \quad if \quad \quad m>\omega ~ or~ a_t = buy~ with~ I_{current} < \alpha \\ 0,\quad \quad if \quad \quad m \le \omega , ~ a_t = buy~ with~ I_{current} \ge \alpha ~ or~ a_t = hold\\ PnL_k,\quad \quad if \quad \quad m \le \omega , ~ a_t = sell,\\ \end{array} \right. \end{aligned}$$
  In the equation above, when the action is buy or sell, *m* becomes $m+1$. This formula provides a way to measure the instantaneous reward obtained at each time step, given the current state of the investment, the cost of the transaction, and the predefined risk threshold. To determine the optimal threshold, three different risk levels are considered, whereby the agent is trained separately for each level: 30% (low), 55% (medium), and 80% (high)^56^. The performance of the agent under these different risk levels is analyzed and reported in “Experiment and results” section. Furthermore, as the Bitcoin market operates 24/7 and our dataset reported hourly, the maximum number of possible active trades was 24 per day. To explore the optimal number of trades for the proposed model, we defined three different thresholds: up to 8, 16, and 24 active trades per day. By testing these thresholds, a better understanding can be acquired on how the agent’s trading activity impacts performance. The results of the experiments conducted with these thresholds are presented in “Experiment and results” section, showcasing the effectiveness of the proposed trading strategy under various levels of trading activity.

In incorporating these factors into the reward function, the goal is to create an agent capable of making effective trading decisions that balance risk, profitability, and trading activity, ultimately for an optimal trading strategy. The key components and layers of the architecture used in our trading strategy are outlined, explaining the role of each layer in extracting meaningful information from the input data and estimating the action-value function *Q*(*s*, *a*). The model architecture begins with an input layer that has two neurons which capture the relevant information in the data, necessary for making trading decisions. These neurons represent market actions (− 1, 0, or 1) and the price prediction score (ranging from − 100 to 100).

The DQN model contains three fully connected dense layers, each containing 64 neurons. These layers aim to capture the complex relationships between the input features and actions. Increasing the number of neurons and layers allows the model to learn more complex patterns, at the expense of increased computational cost and risk of overfitting. Each dense layer utilizes a ReLU activation function to prevent vanishing gradient issues during training. The choice of the activation function is critical, as it introduces nonlinearity into the model, enabling the learning of intricate patterns.

The output layer of the model comprises three neurons corresponding to the three possible actions: buy, sell, or hold. These neurons represent the Q-values for each action, given the current state, and help the model produce a probability distribution for the actions to guide the decision-making process. To minimize the difference between the predicted and target Q-values, the model uses the MSE loss function. This choice of the loss function dictates model learning from errors and parameter updates. The Adam optimizer was employed in this model because it strikes a balance between fast convergence and stability during training.

## Experiment and results

In this section, the results of the experiments conducted to determine the effectiveness of the reward function, risk level, and active trading thresholds are presented for the proposed Main-DQN model and a Bitcoin trading task. To conduct the experiments, we created an environment using Python, utilizing the Pandas library for data preprocessing and employing TensorFlow and Keras for model training and testing, respectively. To train and test the Main-DQN model, the dataset was split into two parts. The model was trained on a dataset comprising trading decisions and price predictions, with the data spanning the period of October 1, 2014 to October 14, 2018. The Main-DQN model incorporated a deep neural network architecture and a set of hyperparameters fine-tuned to optimize the trading strategy for a given task. The choice of the optimizer influences the speed and stability of the learning process, making it a critical factor in the overall performance of the model. In our experiment, the learning rate was set to 0.001 and the discount factor ($\gamma$) to 0.95. The exploration rate ($\epsilon$) started at 1 and decayed exponentially, with a decay factor of 0.995. Despite differing structures and tasks, the three DQN models share similar hyperparameters and the rationality of this was a deliberate choice. Using a common set of hyperparameters created a consistent baseline for comparison, isolating the impact of each model’s unique architecture.

The target network update frequency in the model was set to 400 steps. This determines how often the parameters of the target Q-network are updated, which affects learning stability. Among the different update frequencies used in the experiment, 400 Hz provided an optimal balance between stability and responsiveness. Finally, the batch size for the experience replay was set to 64. This implies that during training, the agent samples random minibatches of 64 experiences from the replay buffer to update the Q values. Table 3 provides a concise overview of the architectures and hyperparameters used in the three DQN models of this study to determine market actions and sentiment scores. Complementing this detailed presentation, Fig. 6 provides a visual depiction of the experimental procedures and offers a comprehensive presentation of the methodology.

The experimental results are presented in four distinct subsections, each focusing on a different aspect of model performance. (1) First, the various evaluation metrics used to assess the performance of the M-DQN model in the Bitcoin trading task are discussed in terms of PnL and risk-adjusted level. (2) Second, the M-DQN model’s performance is analyzed under different risk levels. By varying the risk thresholds in the reward function, the most suitable risk level for the trading strategy is identified. The impact of varying the number of active trades on the M-DQN model performance is also assessed, with the goal of determining the optimal active trading threshold for the trading strategy. (3) Third, the efficiency of the proposed reward function is compared with two existing reward functions from the literature, to demonstrate the effectiveness of the proposed approach. (4) Finally, the results are compared with those of other state-of-the-art studies in the field of trading strategy optimization. This comparison further validates the performance of the M-DQN model and its potential applicability to real-world trading tasks.

**Table 3:** Summary of the M-DQN model architecture and hyperparameters for the Bitcoin trading task, incorporating market action and sentiment scores.

Component	Description	Trade-DQN	Predictive-DQN	Main-DQN
Input layer	Input neurons for market action and sentiment score	1 neuron	2 neurons	2 neurons
Dense layer	Fully connected layers with ReLU activation function	3 layers (64, 32, 8 neurons each)	3 layers (64 neurons each)	3 layers (64 neurons each)
Output layer	Output neurons for representing all possible actions	3 neurons	20,001 neurons	3 neurons
Loss function	Metric to quantify the difference between predicted and true values, guiding weight update function	MSE	MSE	MSE
Optimizer	Algorithm used to update and compute network weights	Adam	Adam	Adam
Learning rate	Step size at each iteration while moving toward a minimum of the loss function	0.001	0.001	0.001
Discount factor	Factor by which future rewards are diminished compared to immediate ones	0.95	0.95	0.95
Exploration rate	Probability of selecting a random action rather than the model choice	1	1	1
Exploration decay	Rate at which exploration decreases over time	0.995	0.995	0.995
Target network update	Frequency of updating the target Q-network’s parameters	Every 400 steps	Every 400 steps	Every 400 steps
Batch size	Batch size for experience replay	64	64	64

By presenting the experimental results in a comprehensive manner, we aim to provide a thorough evaluation of the effectiveness of the M-DQN model in optimizing Bitcoin trading strategy, considering various factors such as risk, return, and active trading.

### Performance measures

Evaluation metrics play a crucial role in assessing the performance of a trading strategy, as they provide quantitative measures for gauging effectiveness in different aspects. Through an analysis of these metrics, the strengths and weaknesses of the strategy can be identified, enabling us to refine and optimize the model to achieve better results in real-world trading scenarios.

(1): *Return of Investment (ROI)* This is one of the primary evaluation metrics used in this study, representing the net gain or loss of an investment after the completion of a trade^57^. ROI is an essential measure, as it directly captures the financial outcome of a trading strategy, indicating profit or loss. In our experiment, ROI was calculated as follows:
  5 $$\begin{aligned} {ROI} =\frac{Final\ cash-Initial\ cash}{Initial\ cash}*100\%. \end{aligned}$$
  Here, *Initial* *cash* is the amount of investment at the beginning, and *Final* *cash* is the amount of investment at the end of the trading. By calculating the ROI of the trade and analyzing it, the overall profitability of the trading strategy can be determined.
(2): *Sharpe Ratio (SR)* SR is a widely used metric in finance for assessing the risk-adjusted performance of an investment or trading strategy. In the context of Bitcoin trading, SR measures the average return generated by the trading strategy, relative to the risk-free rate, per unit of risk, as represented by the standard deviation of the returns. The higher the SR, the better is the risk-adjusted performance of the trading strategy. Amjad et al.^58^ proposed an equation to calculate the SR for Bitcoin trading, letting *N* denote periods; $profit_i$ denote the profit (or loss) achieved during the $i^{th}$ period, where $1\le i\le N$; and $p_0$ and $p_N$ denote the price of Bitcoin at the start and end of the given interval (i.e., *N* periods), with SR determined as follows:
  6 $$\begin{aligned} {SR} =\frac{\frac{1}{N}\sum _{i=1}^{N}{profit_i}-\left| p_0-p_N \right| }{\frac{1}{N}\sum _{i=1}^{N}{profit_i}^2-\left( \frac{1}{N}\sum _{i=1}^{N}{profit_i} \right) ^2}. \end{aligned}$$
  In practice, an SR between 1 and 2 is considered good. A ratio between 2 and 3 is very good, and any result >3 is excellent.

### Experiment on different thresholds $\alpha$ and $\omega$

Before discussing the experimental results, it is necessary to define the two terms used in this subsection to demonstrate the effectiveness of the preprocessing DQN.

(a): *Proposed method* In the proposed method, the Main-DQN model leverages the previously described dataset of “Multi-level DQN” section, consisting of a two-dimensional array with elements taken from the outputs of Trade-DQN and Predictive-DQN models. The preprocessing DQN aims to better extract and capture the relationship between Bitcoin prices and Twitter sentiment scores.
(b): *Classic method* By contrast, in the classic method, the Main-DQN model directly utilizes raw data, including Bitcoin historical prices and Twitter sentiment scores, without any preprocessing DQN or additional data transformations. This means that the Main-DQN model relies solely on the raw data to capture the relationship between the variables.

In defining these methods, the goal is to highlight the potential benefits of the preprocessing DQN, as opposed to using the raw data directly, in enhancing the performance of the Main-DQN model for the development of effective Bitcoin trading strategies.

Subsequent to Main-DQN model training, we evaluated the performance using the remaining portion of the dataset. This test set comprised 30 days (720 h) of data covering the remaining part of the dataset.

**Table 4:** Comparison of classic and proposed methods with varying thresholds at an adjusted 30% level of risk.

Active trade threshold $$\omega$$ (per day)	Initial investment ($)	Number of trades	Final investment ($)	ROI (%)	SR
Classic method (risk rate $\alpha$ - 30%)					
Up to 8 times	1,000,000	167	1,037,293	3.7	2.628
Up to 16 times	1,000,000	283	1,128,492	12.8	2.634
Up to 24 times	1,000,000	492	1,098,814	9.8	2.631
Proposed method (risk rate $\alpha$ - 30%)					
Up to 8 times	1,000,000	207	1,092,843	9.2	2.737
Up to 16 times	1,000,000	325	1,146,942	**14.6**	**2.739**
Up to 24 times	1,000,000	544	1,108,284	10.8	2.737

Significant values are in bold.

As previously mentioned, the proposed reward function was designed to focus on the three primary factors of an efficient trading strategy. To strike a balance between these factors, three distinct thresholds were established for risk management by varying the allowed risk levels: 30% (low), 55% (medium), and 80% (high). Three other thresholds were defined for active trading by varying the number of permitted active trades per day: up to 8, 16, and 24 times. The Main-DQN model was trained separately for each case, and the ROI and SR values were obtained. The experimental results depict these values, whereby the most suitable thresholds were selected through comparisons in each case.

First, the M-DQN model was tested by defining the risk threshold ($\alpha$) as 30%. In Table 4, the results show that the highest ROI for the classic method is observed when the active trading threshold ($\omega$) is set to 16 times per day, yielding a 12.8% ROI. In terms of risk-adjusted returns, the SR values for the classic method are relatively close, but the maximum value is 2.63424, demonstrating a reasonable level of return, given the level of risk involved. Similarly, the proposed method achieves the highest ROI of 14.6%, with the same active trading threshold of 16 times per day. The SR values obtained using the proposed method also exhibit a slight difference, with a maximum value of 2.73962. This further reinforces that the proposed method provides not only a higher return but also a slightly more favorable risk-return tradeoff, as indicated by the higher SR value compared to that of the classic method.

To further explore, the threshold was raised for the permissible level of risk that the agent could take. In this scenario, the agent was allowed to undertake trading risks of up to 55% of the investment without incurring any penalties. If the investment status fell below 45%, the agent received a penalty in the form of a negative reward. As in the previous experiment, three different thresholds were implemented for the number of active trades to identify the optimal performance under the current risk level. Table 5 presents the experimental results. In the experiment, the highest ROI values were achieved when the active trade threshold was set to 16 times per day for both the classical and proposed methods. The ROI values were 23.5% for the classic method and 29.9% for the proposed method, further emphasizing the advantages of the proposed method over the classic method. As the level of allowed risk was increased, lower SR values than those of the previous experiment were anticipated. In both the classic and proposed methods, the SR value is higher than 2.2. Nonetheless, the proposed method outperforms the classic method in terms of risk-adjusted returns, with an SR of 2.39421.

**Table 5:** Comparison of classic and proposed methods with varying thresholds at an adjusted 55% level of risk.

Active trade threshold $$\omega$$ (per day)	Initial investment ($)	Number of trades	Final investment ($)	ROI (%)	SR
Classic method (risk rate $\alpha$ - 55%)					
Up to 8 times	1,000,000	218	1,134,521	13.4	2.284
Up to 16 times	1,000,000	403	1,235,356	23.5	2.315
Up to 24 times	1,000,000	692	1,108,432	10.8	2.299
Proposed method (risk rate $\alpha$ - 55%)					
Up to 8 times	1,000,000	227	1,134,558	13.4	2.324
Up to 16 times	1,000,000	372	1,299,381	**29.93**	**2.394**
Up to 24 times	1,000,000	708	1,192,961	19.2	2.347

Significant values are in bold.

Finally, to assess the performance of the M-DQN model under a high level of risk, the risk threshold was increased to 80%. In this scenario, the agent was permitted to take higher risks to maximize its reward, which translates into higher profits. However, it is important to note that high risk can lead to losses in many cases. Similar to the previous experiments, Table 6 displays the results of the M-DQN model trained with different active trading thresholds considering an 80% risk level. Examining the data in Table 6, we draw several conclusions. First, the optimal threshold for active trading remains up to 16 times per day in all cases. Both the classical and proposed methods achieve their highest ROI at this level, with values of 24.1% and 28.1%, respectively. Second, the findings reinforce the notion that a higher risk level does not always yield a higher profit. This sometimes results in lower profits than those with lower risk levels. Interestingly, as the allowed risk level is increased to 80%, the SR values, which measure risk-adjusted return, are understandably lower than those of the previous experiments. In this case, the classical method yields an SR of 1.58003, whereas the proposed method yields an SR of 1.88034. Despite the higher risk level, the proposed method outperforms the classic method in terms of risk-adjusted returns.

**Table 6:** Comparison of classical and proposed methods with varying thresholds at an adjusted 80% level of risk.

Active trade threshold $$\omega$$ (per day)	Initial investment ($)	Number of trades	Final investment ($)	ROI (%)	SR
Classic method (risk rate $\alpha$ - 80%)					
Up to 8 times	1,000,000	225	1,149,738	14.9	1.507
Up to 16 times	1,000,000	468	1,241,620	24.1	1.580
Up to 24 times	1,000,000	714	1,165,843	16.5	1.539
Proposed method (risk rate $\alpha$ - 80%)					
Up to 8 times	1,000,000	231	1,239,486	23.9	1.817
Up to 16 times	1,000,000	468	1,281,183	**28.1**	**1.880**
Up to 24 times	1,000,000	711	1,257,433	25.7	1.861

Significant values are in bold.

**Figure 7:**
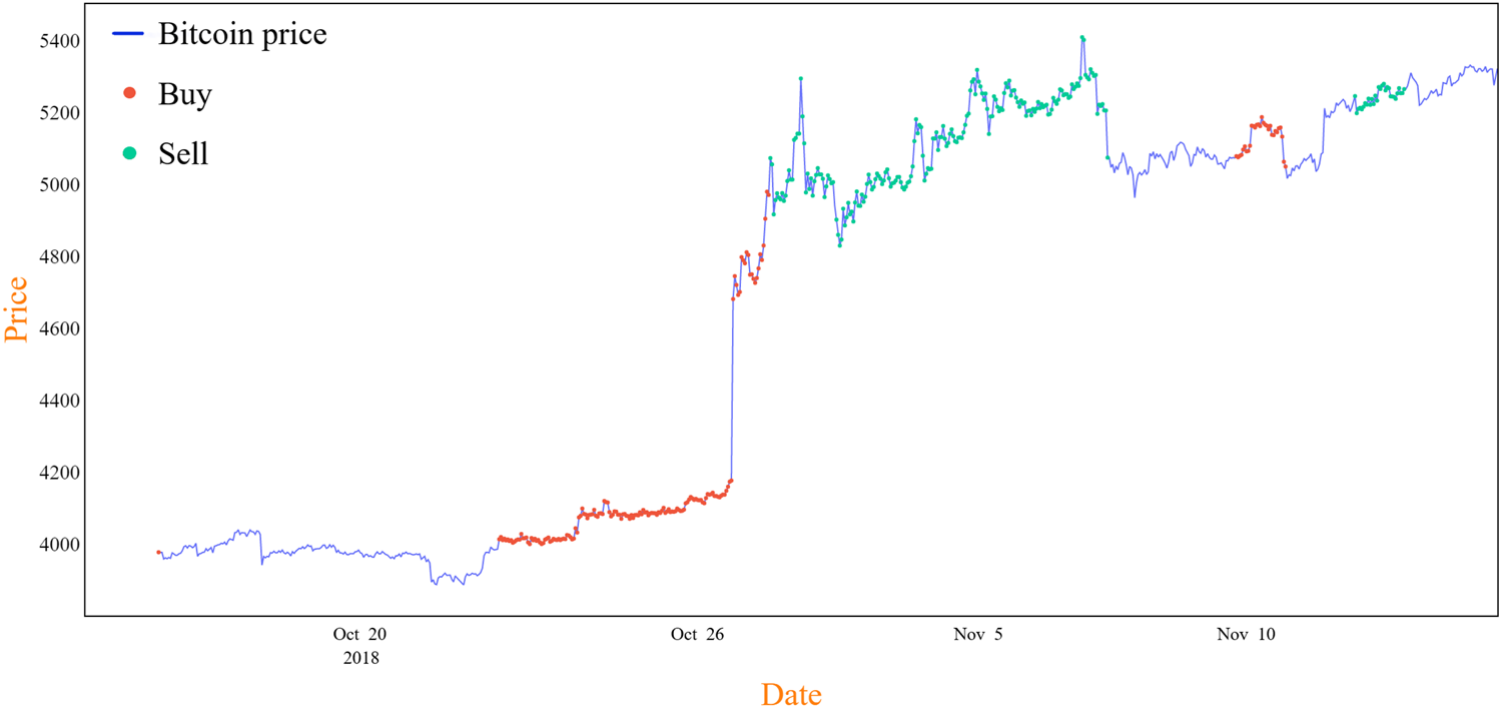
Visualization of the proposed M-DQN agent’s trading strategy with fixed thresholds: 55% of risk level and active trading of up to 16 times per day. The blue line represents Bitcoin price trends; the red dots indicate instances of “buy” action; and the green dots indicate instances of “sell” action.

Finally, the proposed method consistently had higher ROI values than the classical method, further highlighting the efficiency of the proposed preprocessing technique and reward function in enhancing the trading strategy. Figure 7 provides a deeper understanding of the trading process, specifically, with fixed thresholds that yield the most reliable results. In this case, the thresholds are set at a risk level of 55% and a maximum of 16 trades per day. The x-axis in Fig. 7 represents the time span of the experiment, and the y-axis corresponds to the Bitcoin price. The blue line graphically depicts the price of Bitcoin over the duration of the experiment. The red dots indicate instances of the Main-DQN agent deciding to buy Bitcoin, whereas the green dots indicate the instances the agent decides to sell. Upon visual examination, it is observed that the M-DQN model typically purchases Bitcoins at lower prices and sells them after the Bitcoin price significantly increases. This behavior demonstrates the effectiveness of the model in navigating the Bitcoin market.

### Experiments with different reward functions

In this subsection, we present the experimental results of evaluating the performance of the proposed reward function by comparing it with two other reward functions in the literature. The objective of this comparison is to demonstrate the effectiveness of the proposed reward function in the context of Bitcoin trading, thereby highlighting its advantages over alternatives. The reward functions proposed by Allen et al.^59^ and Sadighian^60^ was selected as the benchmark for this comparison.

Allen et al.^59^ indicated that excess returns can be calculated in two ways based on the action type. For buy or sell action, the return is computed as follows:

7 $$\begin{aligned} {r_t} =\sum _{t=1}^{T}(\log P_t-\log P_{t-1})+\sum _{t=1}^{T}r_f+n\log \left( \frac{1-c}{1+c}\right) . \end{aligned}$$

where *P* is the daily closing price for a given day *t*; *c* denotes the one-way transaction cost; $r_f$ is the risk-free cost; and *n* denotes the number of trades. If the action is to wait (hold), it is computed as:

8 $$\begin{aligned} {r_t} =n\log \left( \frac{1-c}{1+c}\right) . \end{aligned}$$

The second benchmark was the reward function proposed by Sadighian^60^. In this study, seven different reward functions were proposed and their impact on an agent’s market-making strategy was evaluated. Overall, the trade completion (TC) reward function generated the highest return, and TC was computed as follows:

9 $$\begin{aligned} {TC_t=} \left\{ \begin{array}{l} {1}, \quad \quad \quad \quad \quad if\quad RPnL_t^{step} \ge 2\varpi \\ {-1} \quad \quad \quad \quad \quad if\quad RPnL_t^{step} \le -\varpi \\ {RPnL_t^{step},} \quad \quad otherwise. \end{array} \right. \end{aligned}$$

where $\varpi$ is the transaction fee, and $RPnL_t^{step}$ is the realized profit or losses obtained between time steps *t* and $t-1$ and is calculated as

10 $$\begin{aligned} {RPnL_t^{step}} =\left[ \frac{Ex_t^{E,short}}{Ex_t^{X,cover}}-1 \right] +\left[ \frac{Ex_t^{X,sell}}{Ex_t^{E,long}}-1 \right] . \end{aligned}$$

Here, $Ex_t^{E,short}$ and $Ex_t^{E,long}$ are the average entry prices for the long and short sides, respectively, and $Ex_t^{X,sell}$ and $Ex_t^{X,cover}$ are the average exit prices of the actions executed between time steps *t* and $t-1$. The Main-DQN model was trained separately based on the two reward functions, thereby obtaining both ROI and SR values. By analyzing each value obtained from the Main-DQN models trained with each reward function, the performance of the proposed reward function was assessed in comparison to the alternatives. This comparison enabled us to determine the superiority of the proposed reward function in terms of better trading strategies and overall performance. Table 7 shows the ROI and SR values for each reward function. The performance of the proposed reward function is the best with the highest values as in the previous experiments, for both the classical and proposed methods.

**Table 7:** Comparison of classical and proposed methods for different reward functions.

Reward function	Initial investment ($)	Number of trades	Final investment ($)	ROI (%)	SR
Classic method					
Allen et al.^59^	1,000,000	147	1,110.968	11.0	1.903
Sadighian^60^	1,000,000	209	1,116,987	11.6	1.954
Ours	1,000,000	403	1,235,356	23.5	2.634
Proposed method					
Allen et al.^59^	1,000,000	185	1,151,961	15.1	2.113
Sadighian^60^	1,000,000	242	1,166,377	16.6	2.209
Ours	1,000,000	372	1,299,381	**29.93**	**2.739**

Significant values are in bold.

The data presented in Table 7 offer several observations. In the classic method, the proposed reward function achieves the highest ROI of 23.5.

Similarly, for the proposed method, the proposed reward function exhibits superior performance, yielding the highest ROI of 29.9%. This underscores not only the effectiveness of the proposed reward function for both the classic and proposed methods but also the inherent advantage of the proposed method. This is further supported by an even stronger SR of 2.73962, which indicates a robust risk-adjusted return, despite a higher risk level.

### Performance comparisons

To thoroughly assess the effectiveness of the proposed M-DQN model, we examine the results that demonstrate the profitability and risk level of the trading strategies, using ROI and SR as metrics, respectively. We compared these outcomes with those of similar studies that employ different methodologies for efficient trading strategies, to obtain a better understanding of the performance of the proposed model. This comparative analysis not only enabled us to pinpoint the areas in which the proposed model excels but also helped identify the aspects that may need further improvement. Through this evaluation, valuable insights were acquired into the robustness of our approach and its potential for real-world applications in trading strategies. The results of previous studies are compared with those of the proposed method in Table 8.

In Table 8, we have provided a performance comparison of our M-DQN model against several notable methodologies in the domain of cryptocurrency trading. This comparison is essential to demonstrate the effectiveness and innovation of our approach to existing strategies. We included the DNA-S method by Betancourt et al.^61^, which offers a unique perspective in algorithmic trading, and the Sharpe D-DQN by Lucarelli et al.^62^, a variation of the DQN model emphasizing the Sharpe ratio. Additionally, we compared our model with the Double Q-network combined with a Beep Boltzmann Machine by Bu et al.^63^, showcasing an integration of Double Q-learning with Boltzmann Machines. For a direct comparison, we also included the standard DQN approach by Theate et al.^64^. Furthermore, our analysis extends to the TD3 model by Majidi et al.^65^, which employs continuous action space in deep reinforcement learning, and a forecasting model by Amjad et al.^58^, which leverages econometric approaches for Bitcoin price prediction. This diverse range of comparisons, encompassing various machine learning paradigms, was deliberately chosen to provide a thorough evaluation of our model’s performance against both traditional and innovative approaches in the field.

**Table 8:** Performance comparison with other results.

Metrics	Studies	Year	Value
ROI	DNA-S (Betancourt et al.^61^)	2021	> 24%
	SharpeD-DQN (Lucarelli et al.^62^)	2019	26.14%
	Double Q-network with Beep Boltzman Machine (Bu et al.^63^)	2018	27.87%
	DQN (Theate et al.^64^)	2021	29.4%
	TD3 (Majidi et al.^65^)	2022	57.5%
	M-DQN (our paper)	2023	29.93%
SR	TD3 (Majidi et al.^65^)	2022	1.53
	Forecasting model (Amjad et al.^58^)	2017	> 2.0
	M-DQN (our paper)	2023	2.74

The “>” symbol in the table indicates greater than the corresponding number.

As a result, M-DQN first outperforms the other models in terms of SR, achieving a value of 2.73962. This indicates that the model can generate higher risk-adjusted returns than those used in other studies. However, the ROI metric, indicating model performance, ranks second with a value of 29.93%. The highest ROI was reported by Majidi et al.^65^, who obtained a 57.5% ROI value. Despite having the lowest performance in terms of ROI, the proposed model has a significantly higher SR value (1.53) than that of Majidi et al.^65^. Because the SR metric is used to describe the risk level, with higher values indicating less risk, we concluded that the strategy developed by the proposed M-DQN model is less risky and more robust than that of other models. A higher SR value suggests that the model generates more consistent and stable returns, making it a more attractive choice for investors who prioritize risk management.

## Discussion

This study demonstrated that it is more effective and resource-efficient to extract and utilize data tailored for price prediction than using all available data when developing an effective Bitcoin trading strategy. Specifically, by incorporating both market actions and sentiment scores derived from a set of influential Twitter users, the proposed method yielded significantly better results than the classical method which uses all available raw data. Moreover, the proposed model contributes to the literature in designing a reward function for DQN agents involved in Bitcoin trading and reinforcement learning, offering valuable insights for further in-depth analysis.

Nevertheless, it’s important to recognize that the methodology introduced in this study comes with certain limitations and corresponding future works that warrant discussion.

(1): * Diversity of data* First and foremost, our model chiefly relies on a combination of market action data and Twitter sentiment scores to make projections about Bitcoin prices. These metrics, although significant, may not adequately capture the multitude of variables at play in shaping Bitcoin’s fluctuating market value. Twitter is indeed a resource-rich platform for sentiment analysis, but it is not the only platform where financial discussions take place. Hence, our future work could focus on a more rounded approach that could involve examining other social media platforms, such as Reddit and Facebook. Furthermore, mainstream news sources including YouTube broadcasts and traditional television news channels might offer a fuller, multi-dimensional view of market sentiment that is not currently accounted for in our model. This consideration would allow us to capture people’s opinions with far more accuracy than potentially leading an accurate predictions.
(2): * Diversity of ML models* Second, the trading model under examination employs a DQN algorithm as its core predictive mechanism. DQN algorithms have exhibited efficacy in a range of applications, but they are not the end-all-be-all of machine learning solutions. It is also worth noting that the hyperparameters deployed in the current DQN models are based on commonly accepted values. While these parameters have proven effective, there is room for further optimization. Therefore, it is recommended that subsequent research explores expanding the model to incorporate other DRL algorithms, such as Proximal Policy Optimization (PPO), Twin Delayed DDPG (TD3), or Advantage Actor-Critic (A2C), which could introduce added layers of sophistication and flexibility. By doing so, it is conceivable that the model could fine-tune the accuracy of the Q-function, leading to an enhancement in the trading system’s overall predictive capabilities. Moreover, experimenting with different neural network hyperparameters, such as types of functions, batch sizes, and discount factor values—can lead to even better results. In essence, the performance benchmarks set by the current study may serve as a starting point, and fine-tuning the neural network hyperparameters could unlock further improvements in model accuracy.
(3): *Complexity of reward function* The third, yet significant, limitation lies in the structure of the current reward function employed within the DQN algorithm. The existing reward function primarily serves to encourage profitable trading, adjusted risk rate, and active trading, but it may not be sufficiently tailored to address the complexities and nuances inherent in the trading environment. Specifically, the current reward function may overlook important real-world considerations such as transaction costs, slippage, market liquidity, and the impact of large trades on market prices which are critical for evaluating the true effectiveness of trading strategy. To overcome the limitation regarding the reward function, future investigations should consider real-world trading problems such as transaction costs, slippage, market liquidity, and the impact of large trades on market prices, aiming to produce seemingly profitable strategies that are suboptimal when applied to a more realistic trading context. Therefore, future iterations of this model could benefit immensely from the development and integration of a more sophisticated reward function, thereby creating a trading system that is more aligned with real-world complexities.
(4): * Diversity of cryptocurrencies platforms* Lastly, the current research presents a notable limitation in its focus on Bitcoin and the scope of data utilized. This study’s confinement to a single cryptocurrency platform, while providing in-depth insights, potentially overlooks the diverse and dynamic nature of the broader cryptocurrency market. The exclusive use of sentiment data from limited sources may not fully capture the multifaceted sentiment dynamics that influence the cryptocurrency market. This limitation is particularly significant given the rapidly evolving landscape of cryptocurrencies, where factors influencing one platform may differ substantially from those affecting another. Hence, addressing the diversity of cryptocurrency platforms will require an aim to broaden the scope by integrating data scores from a variety of platforms and considering a wider range of cryptocurrencies, such as Ethereum or Ripple. Such an expansion would not only enhance the robustness of the findings but also adapt the developed trading strategies to the diverse and ever-changing cryptocurrency markets. Rigorous exploration of these different avenues is essential. It would likely lead to the refinement and improvement of the methodologies and trading strategies employed in the current study, offering a more comprehensive understanding of the cryptocurrency trading landscape.

## Conclusion

In this study, we presented the M-DQN model, a novel approach for an enhanced Bitcoin trading strategy. This model integrates both market-action data and sentiment scores from influential Twitter users, showcasing its efficacy and resource efficiency over traditional models that utilize unrefined data. The distinctiveness of our M-DQN model is evidenced by its high SR value of over 2.7, indicating a strong risk-adjusted performance. Additionally, the model achieved an impressive ROI of 29.93%, ranking as the second-best in our comparative analysis. These results underline a trading strategy that is not only profitable but also robust and less risky compared to existing methodologies. For example, when we compare the performances with the other contemporary models, the M-DQN outperforms in terms of risk-adjusted value. As a future work, we suggest that further explorations into diverse Deep Reinforcement Learning algorithms and additional sources of sentiment data, encompassing a broader range of cryptocurrencies, could significantly enhance the efficacy and applicability of the trading strategies we’ve developed.

## Data Availability

This work was supported by the National Research Foundation of Korea (NRF) grant funded by the Korean government (MSIT) (No. 2022R1C1C1004590).
